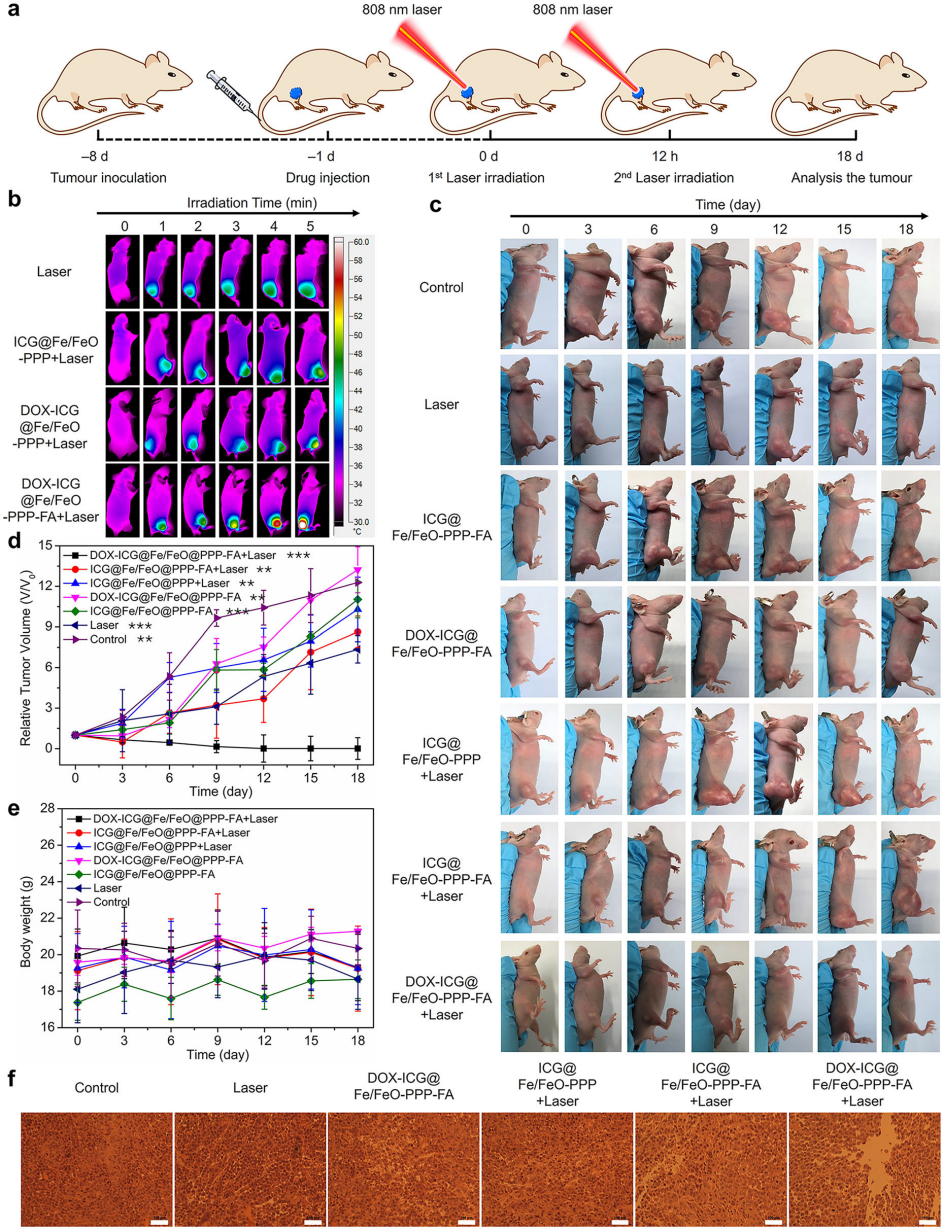# Author Correction: Near-infrared light and tumor microenvironment dual responsive size-switchable nanocapsules for multimodal tumor theranostics

**DOI:** 10.1038/s41467-025-59699-x

**Published:** 2025-05-08

**Authors:** Zhiyi Wang, Yanmin Ju, Zeeshan Ali, Hui Yin, Fugeng Sheng, Jian Lin, Baodui Wang, Yanglong Hou

**Affiliations:** 1 Beijing Key Laboratory for Magnetoelectric Materials and Devices, Department of Materials Science and Engineering, College of Engineering, Beijing Innovation Centre for Engineering Science and Advanced Technology, Peking University, 100871 Beijing, China; 2 State Key Laboratory of Applied Organic Chemistry, Key Laboratory of Nonferrous Metal Chemistry and Resources Utilization of Gansu Province, Lanzhou University, Gansu, 730000 Lanzhou China; 3 College of Life Science, Peking University, 100871 Beijing, China; 4 Department of Radiology, the Fifth Medical Centre, Chinese PLA General Hospital, 100071 Beijing, China; 5 Synthetic and Functional Biomolecules Center, Department of Chemical Biology, College of Chemistry and Molecular Engineering, Peking University, 100871 Beijing, China

Correction to: *Nature Communications* 10.1038/s41467-019-12142-4, published online 27 September 2019

In the version of the article initially published, due to mistakes during figure preparation, there were duplications in Fig. 6c (ICG@Fe/FeO-PPP-FA-0 day, DOX-ICG@Fe/FeO-PPP-FA-18 day, ICG@Fe/FeO-PPP+Laser-12 day, ICG@Fe/FeO-PPP-FA+Laser-6 day) and Fig. 6f (DOX-ICG@Fe/FeO-PPP-FA). The errors were in presentation only and do not affect any conclusions drawn in the study. Due to the age of the paper, the figure cannot be replaced directly; Fig. 6, below, serves to update the article via this amendment.

Fig. 6 Corrected.