# Supplementary material for: Near-infrared light and tumor microenvironment dual responsive size-switchable nanocapsules for multimodal tumor theranostics
Source: Nat Commun. 2019 Sep 27;10:4418. doi: 10.1038/s41467-019-12142-4 (PMC6765052; doi:10.1038/s41467-019-12142-4)
Supplement: Supplementary file 1 — Supplementary Information [file 41467_2019_12142_MOESM1_ESM.pdf]

Supplementary Information

Near-Infrared Light and Tumor Microenvironment Dual Responsive Size-Switchable Nanocapsules for Multimodal Tumor Theranostics

Zhiyi Wang[1,2,†], *Yanmin Ju*[3,†], *Zeeshan Ali1*, *Hui Yin*[4], *Fugeng Sheng*[*,4], *Jian Lin*[5], *Baodui Wang*[*,2], *Yanglong Hou*[*,1]

[1]Beijing Key Laboratory for Magnetoelectric Materials and Devices, Department of Materials Science and Engineering, College of Engineering, Peking University, Beijing Innovation Centre for Engineering Science and Advanced Technology, Beijing 100871, China

[2]State Key Laboratory of Applied Organic Chemistry and Key Laboratory of Nonferrous Metal Chemistry and Resources Utilization of Gansu Province and, Lanzhou University, Gansu, Lanzhou, 730000, China

[3]College of Life Science, Peking University, Beijing 100871, China

[4]Department of Radiology, the Fifth Medical Centre, Chinese PLA General Hospital, Beijing, 100071, China

[5]Synthetic and Functional Biomolecules Center, Department of Chemical Biology, College of Chemistry and Molecular Engineering, Peking University, Beijing 100871, China

Email: fugeng_sheng@163.com; wangbd@lzu.edu.cn; hou@pku.edu.cn

[†]These authors contributed equally.

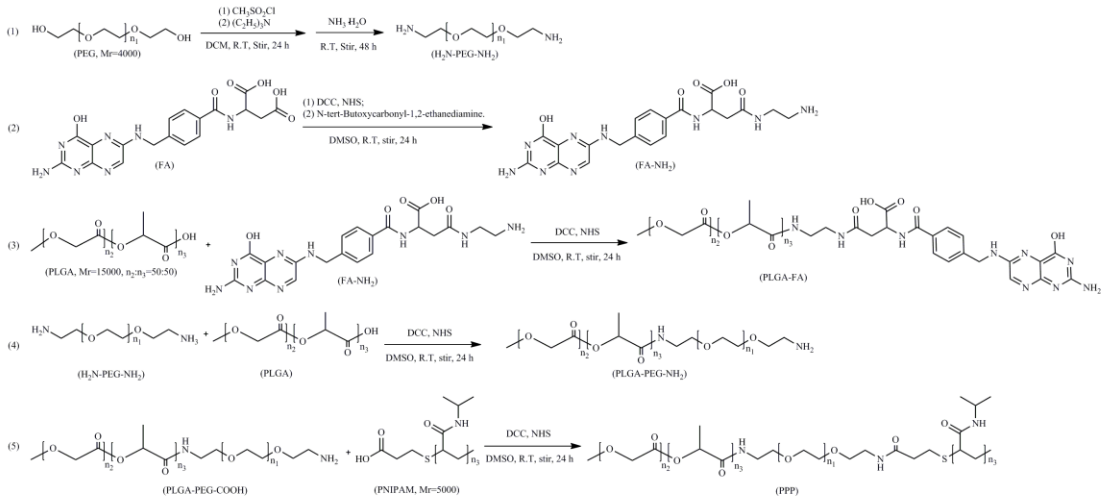

Supplementary Figure 1. Synthetic route of the PPP for fuctional modification. (PPP: PLGA-PEG-PNIPAM.)

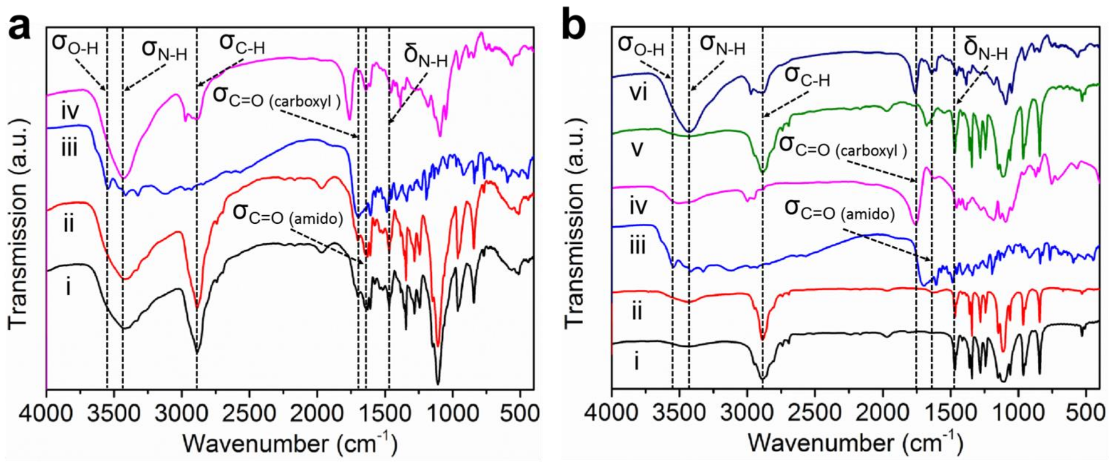

Supplementary Figure 2. FTIR characterization. (a) i: FA, ii: $FA-NH_2$, iii: PLGA, iv: PLGA-FA, (b) i: PEG, ii: $H_2N-PEG-NH_2$, iii: PLGA, iv: PLGA-PEG, v: PNIPAM, vi: PLGA-PEG-PNIPAM.

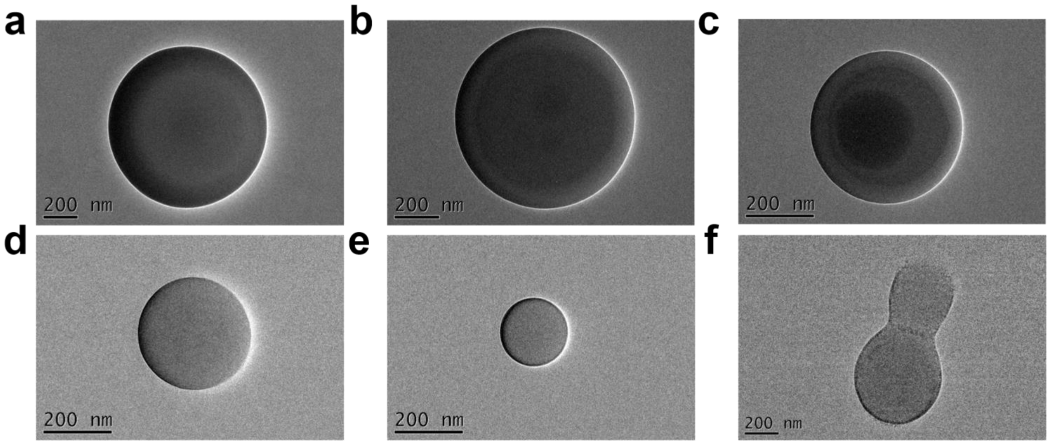

Supplementary Figure 3. TEM image of ICG@PPP nanocapsules in different condition: (a) $V_{dichloromethane}/V_{water}$=1:1, (b) $V_{dichloromethane}/V_{water}$=1:2, (c) $V_{dichloromethane}/V_{water}$=1:4, (d) $V_{dichloromethane}/V_{water}$=1:6, (e) $V_{dichloromethane}/V_{water}$=1:8 and (f) $V_{dichloromethane}/V_{water}$=1:4 and without PVA.

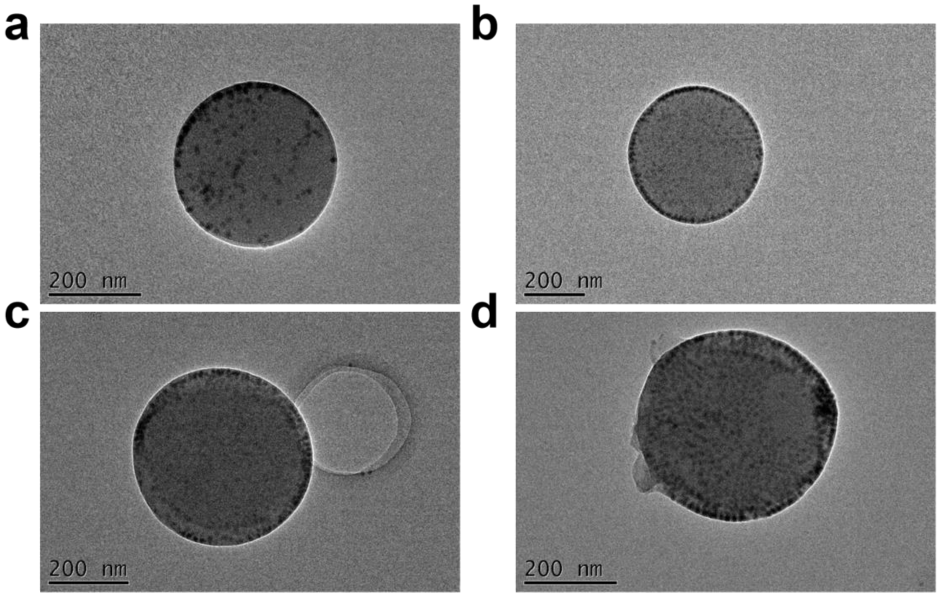

Supplementary Figure 4. TEM image of ICG@Fe/FeO-PPP nanocapsules in different amount of Fe/FeO NCs: (a) $m_{Fe/FeO\ NCs}$=1 mg, (b) $m_{Fe/FeO\ NCs}$=3 mg, (c) $m_{Fe/FeO\ NCs}$=6 mg and (d) $m_{Fe/FeO\ NCs}$=12 mg.

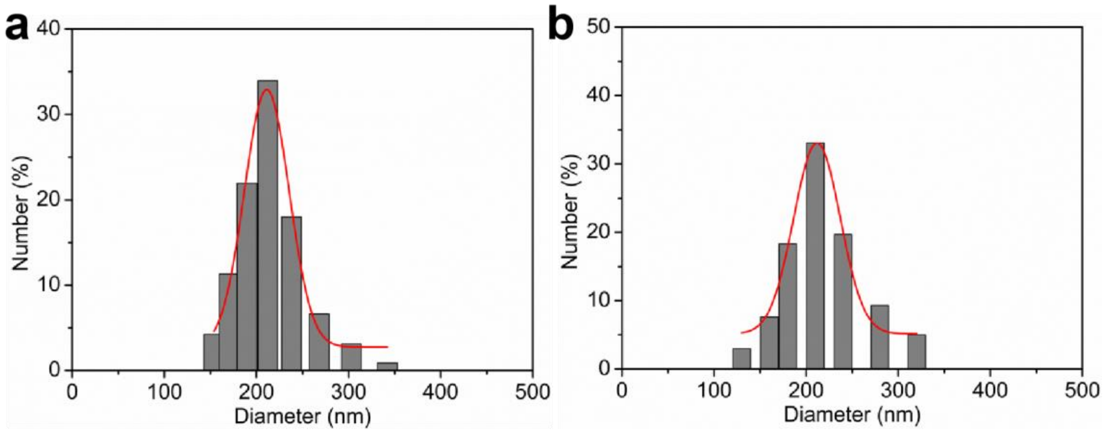

Supplementary Figure 5. Hydrodynamic diameters measured by DLS for the ICG@PPP nanocapsules (203.8±45.7 nm, polydispersity index (PDI)=0.224) (a) and DOX-ICG@Fe/FeO-PPP (218.9±52.1 nm, PDI=0.238) nanocapsules dispersed in in PBS (b).

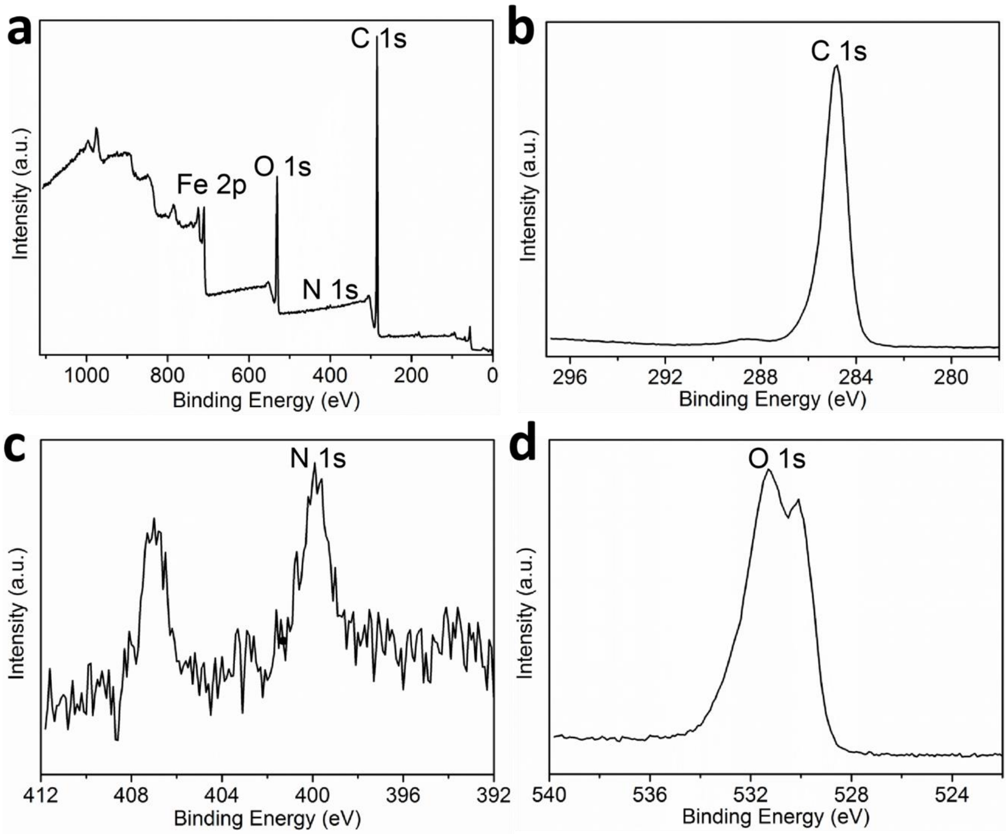

Supplementary Figure 6. XPS of Fe/FeO nanocrystals (a) and High resolution XPS of (b) C 1s, N 1s (c) and O 1s (d).

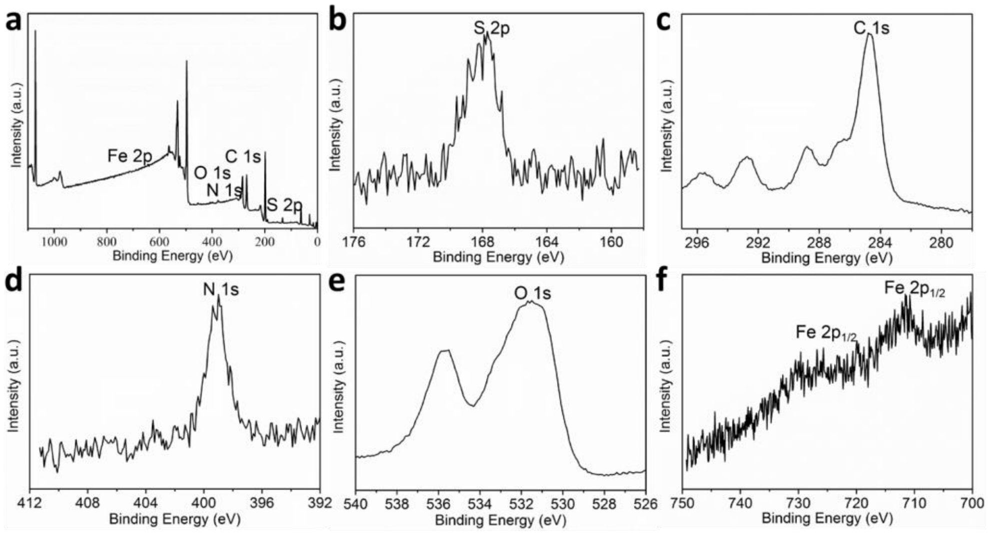

Supplementary Figure 7. XPS of DOX-ICG@Fe/FeO-PPP nanocapsules **(**a**)** and high resolution XPS of S 2p (b**)**, C 1s (c**)**, N 1s (d**)**, O 1s (e**)** and Fe 2p **(**f**)**.

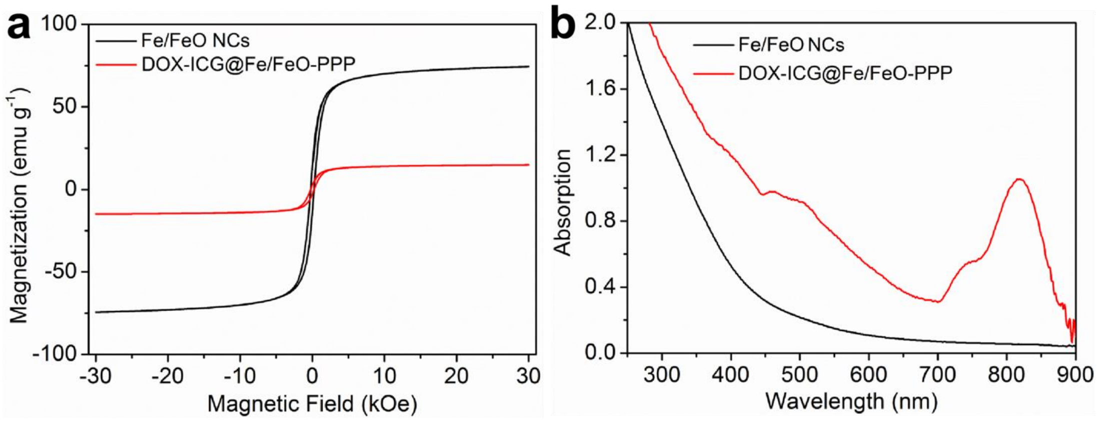

Supplementary Figure 8. (a**)** Magnetic hysteresis loops of the Fe/FeO NCs and DOX-ICG@Fe/FeO-PPP nanocapsules at 298 K. (b) UV-vis absorption spectra of the Fe/FeO NCs and DOX-ICG@Fe/FeO-PPP nanocapsules.

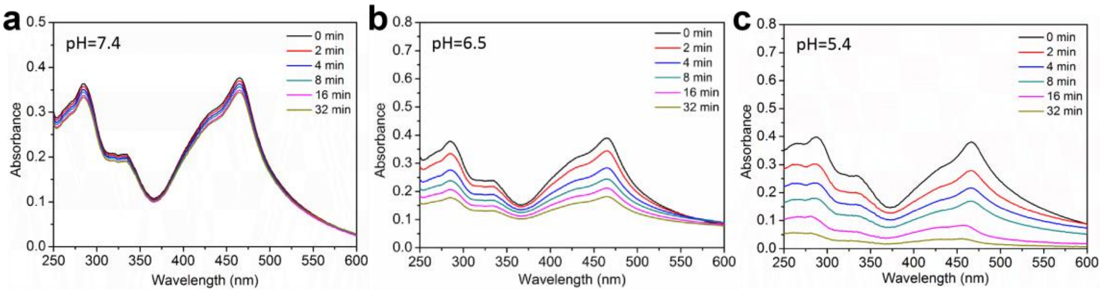

Supplementary Figure 9. UV-vis spectra of DPBF with increasing exposure time for ICG@Fe/FeO-PPP nanocapsules at (a) pH=7.4, (b) pH=6.5 and (c) pH=5.4.

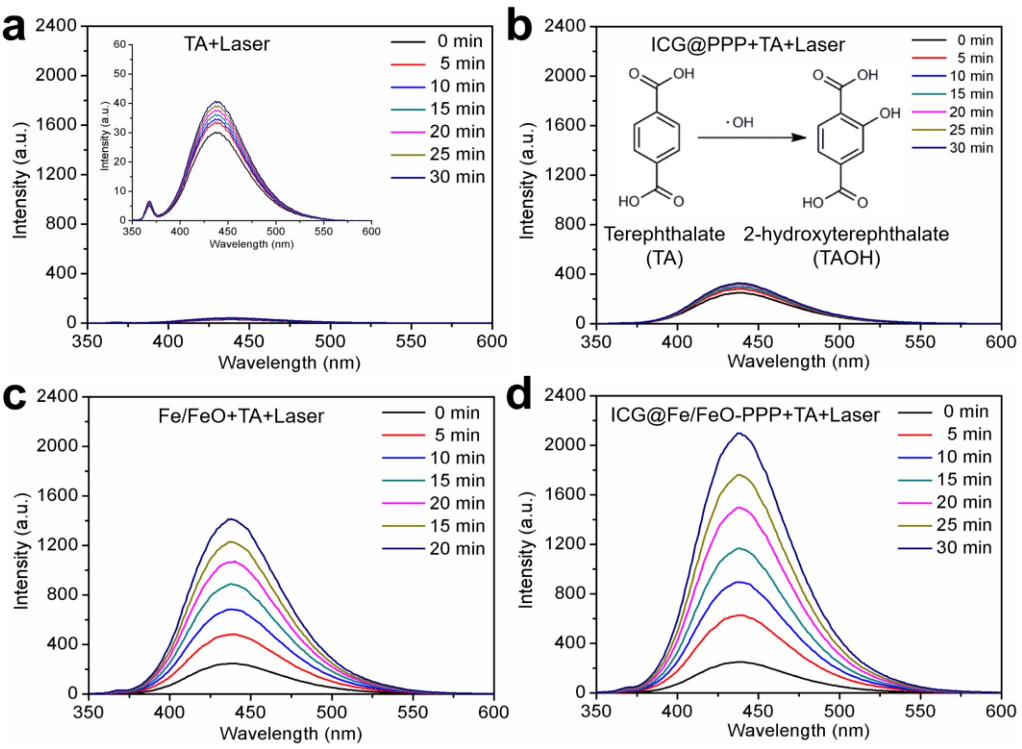

Supplementary Figure 10. Detection of •OH generated by ICG@Fe/FeO-PPP nanocapsules. Fluorescence spectra of TAOH induced by (a) only under 808 nm laser irradiation (0.3 W cm^{-2}), (b) ICG@PPP nanocapsules under 808 nm laser irradiation (0.3 W cm^{-2}), (c) Fe/FeO under 808 nm laser irradiation (0.3 W cm^{-2}) and (d) ICG@Fe/FeO-PPP nanocapsules under 808 nm laser irradiation (0.3 W cm^{-2}) from the same concentration of TA solution for different times (0−30 min). Inset in (b): After the oxidation of terephalic acid (TA) to 2-hydroxy- terephalic acid (TAOH) by •OH, nonfluorescent TA was converted to fluorescent TAOH.

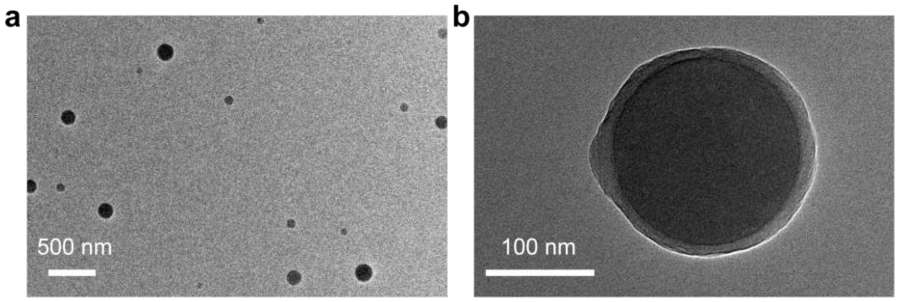

Supplementary Figure 11. TEM image of ICG@PPP-FA nanocapsules in high magnification (a) and low magnification (b).

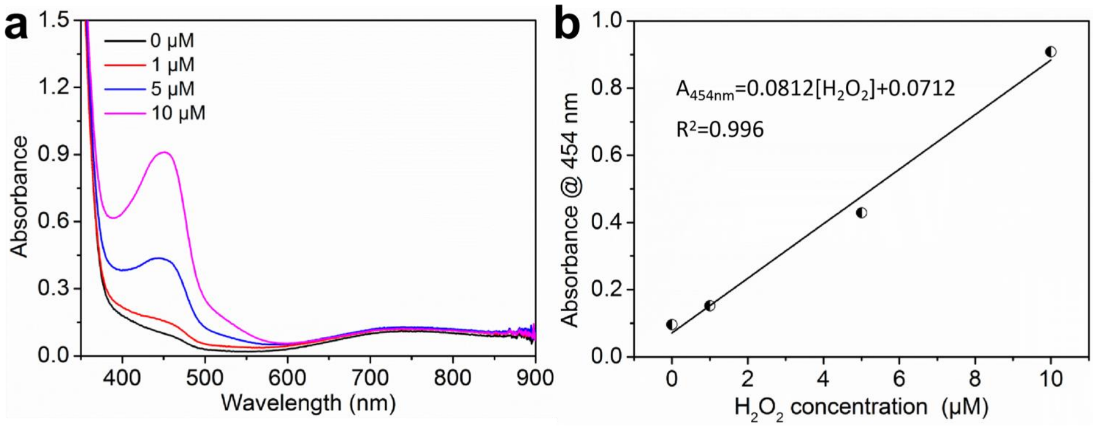

Supplementary Figure 12. Simulation of standard working curve for the detection of H_2O_2 by DMP method. (a) Determination of UV-vis absorbance spectra. (b) Simulation of standard working curve.

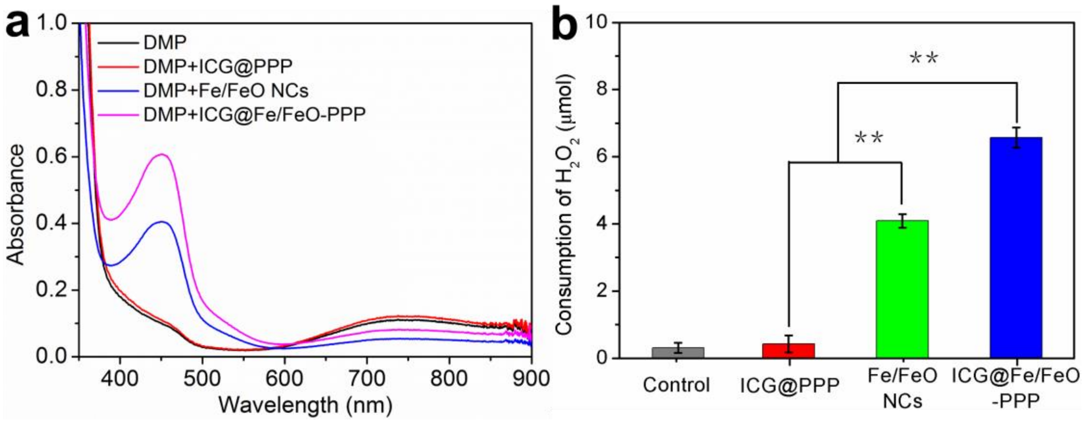

Supplementary Figure 13. (a) UV-vis absorbance spectra of ICG@-PPP nanocapsules, Fe/FeO NCs and ICG@Fe/FeO-PPP nanocapsules for the detection of H_2O_2 by DMP method under the irradiation of 808 nm laser (0.3 W cm^{-2}, 5 min). (b) Detection of •OH by a spectrophotometric method using copper (II) ion and DMP for each nanocapsules group. P values in (b) were calculated by Tukey's post-hoc test (**P<0.01, ***P<0.001) by comparing other groups with the last group (ICG@Fe/FeO-PPP). (Error bars, mean±SD, n=6, DMP: 2,9-dimethyl-1,10-phenanthroline.)

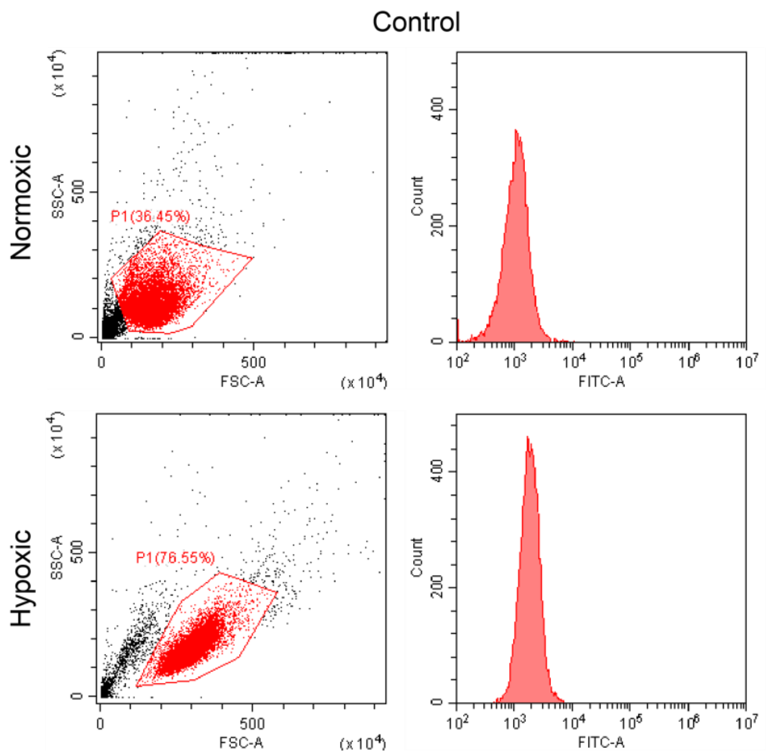

Supplementary Figure 14. $^{1}O_2$ generation evaluated of control group by flow cytometry without DHR123 in KB cells under normoxic and hypoxic condition.

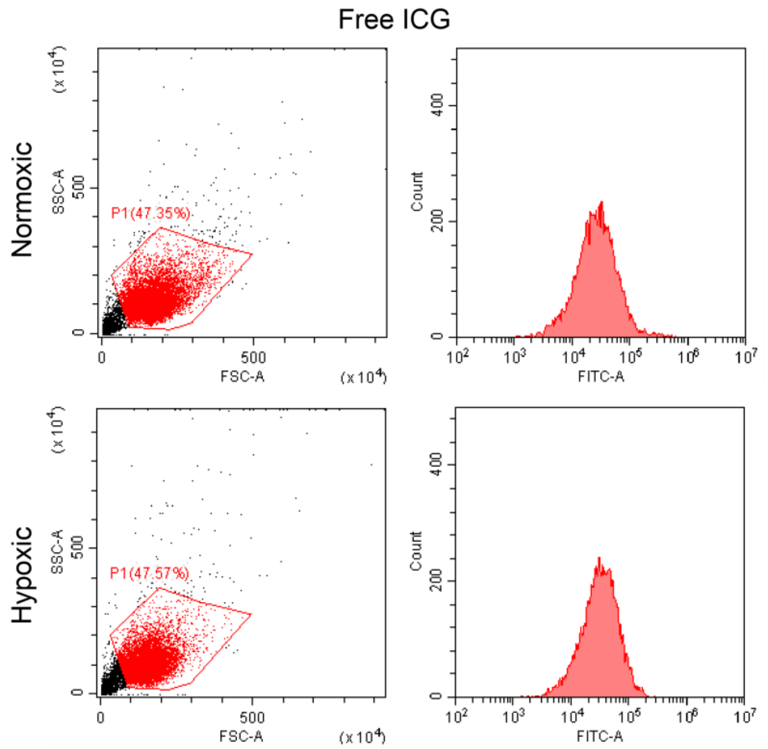

Supplementary Figure 15. $^{1}O_2$ generation evaluated of free ICG group by flow cytometry with DHR123 in KB cells under normoxic and hypoxic condition.

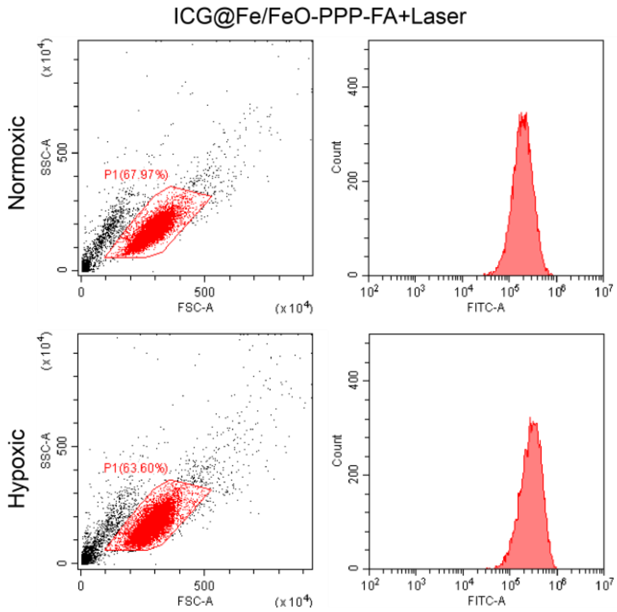

Supplementary Figure 16. $^1O_2$ generation evaluated of ICG@Fe/FeO-PPP nanocapsules group by flow cytometry with DHR123 in KB cells under normoxic and hypoxic condition.

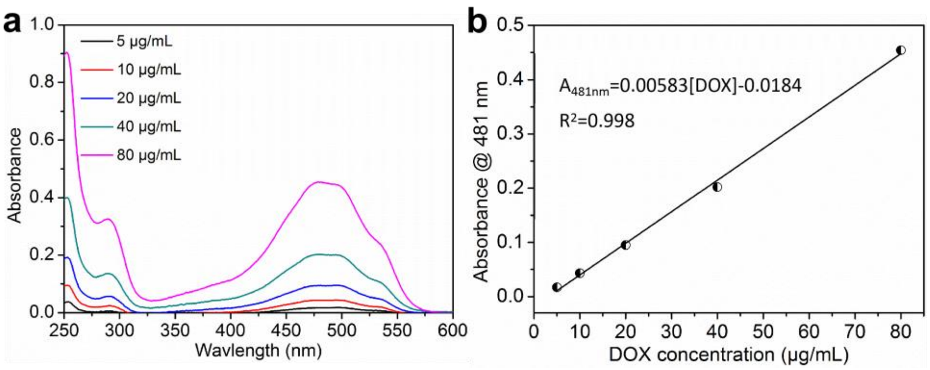

Supplementary Figure 17. UV-vis absorbance spectra (a) and simulation of standard working curve (b) for the detection of DOX.

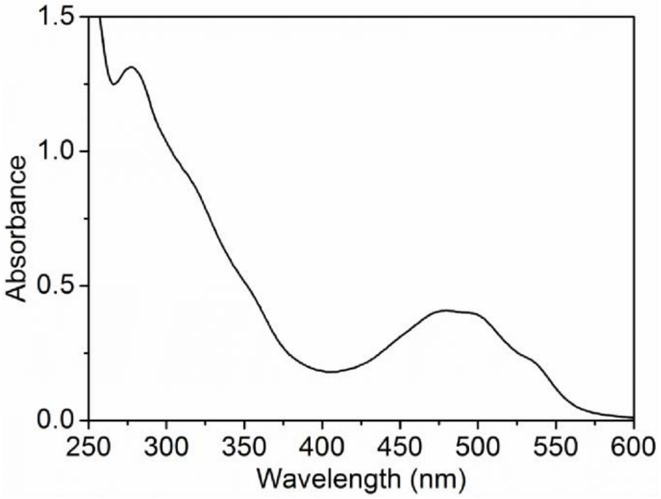

Supplementary Figure 18. UV-vis absorbance spectra of the decomposed DOX-ICG@Fe/FeO-PPP nanocapsules.

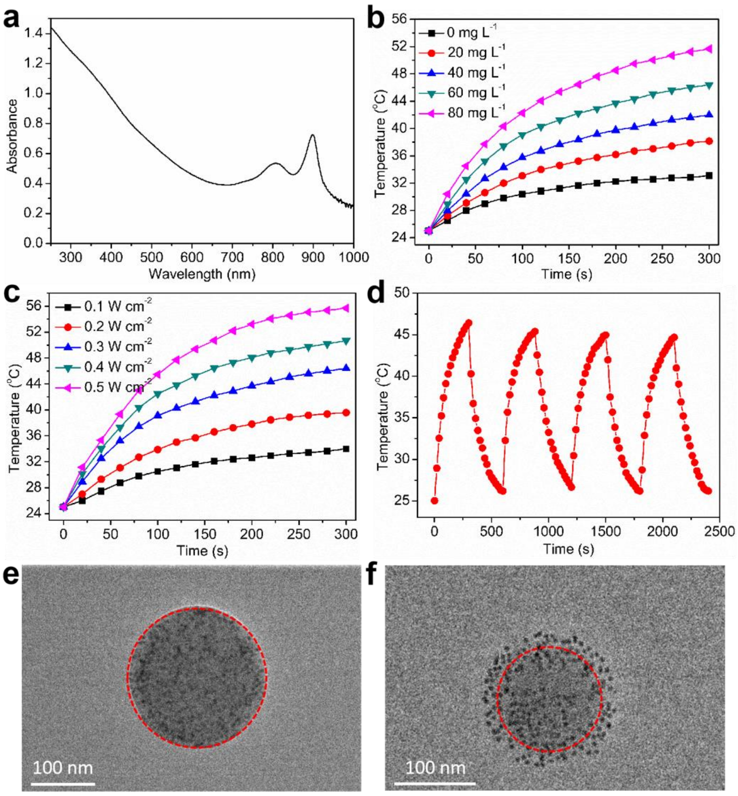

Supplementary Figure 19. (a) UV-vis absorbance spectra of ICG@Fe/FeO-PPP nanocapsules. (b) Temperature curves of ICG@Fe/FeO-PPP nanocapsules dispersions with concentrations of 0, 20, 40, 60 and 80 mg L^{-1} under 808 nm irradiation at a laser power density of 0.3 W cm^{-2} in 5 min. (c)Temperature curves of ICG@Fe/FeO-PPP nanocapsules dispersions with different power densities (0.1, 0.2, 0.3, 0.4, and 0.5 W cm^{-2}) with the concentration of 60 mg L^{-1} under 808 nm laser irradiation in 5 min.; (d) Temperature curves of ICG@Fe/FeO-PPP nanocapsules for four laser on/off cycles under the 808 nm laser with 0.3 W cm^{-2}. TEM image of ICG@Fe/FeO-PPP nanocapsules before (e) and after (f) 5 min irradiation of 808 nm laser in the concentration of 60 mg L^{-1}.

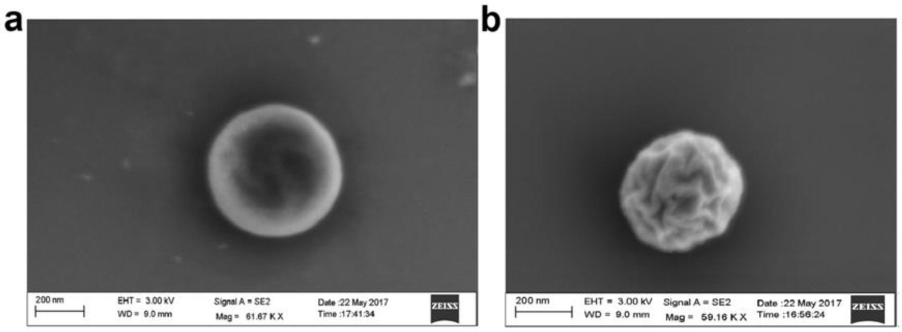

Supplementary Figure 20. SEM image of ICG@Fe/FeO-PPP nanocapsules before (a) and after (b) 5 min irradiation of 808 nm laser (0.3 W cm^{-1}) in the concentration of 60 mg L^{-1}.

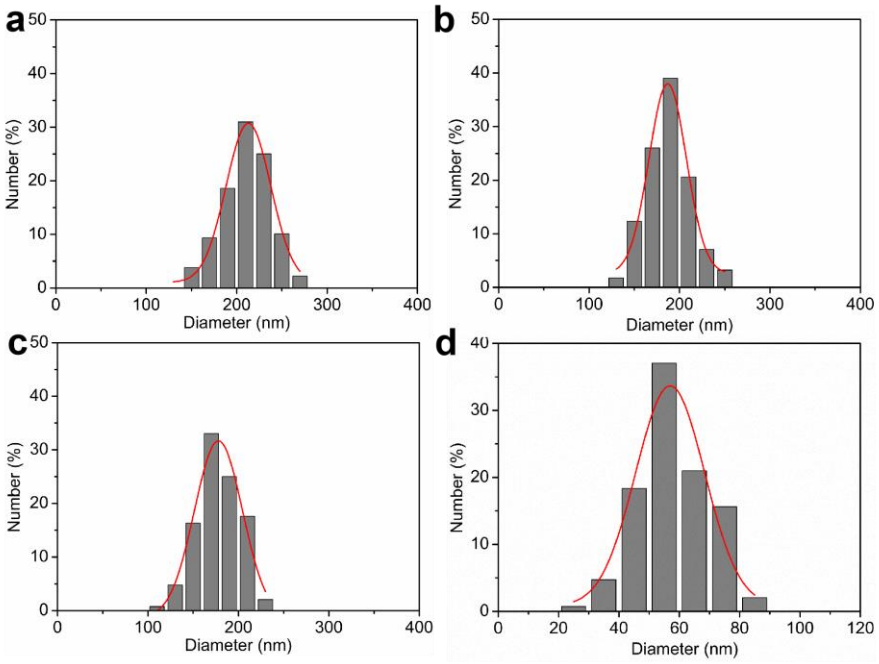

Supplementary Figure 21. Hydrodynamic diameters measured by DLS for the shrinking process of DOX-ICG@Fe/FeO-PPP nanocapsules in (a) 0 h (220.9±25.5 nm, PDI=0.115), (b) 3 h (185.9±28.2 nm, PDI=0.152), (c) 24 h (161.9±21.8 nm, PDI=0.135) and (d) 48 h (54.5±4.1 nm, PDI=0.075) after the irradiation of laser (808 nm, 0.3 W cm^{-2}) for 5 min (pH=6.5).

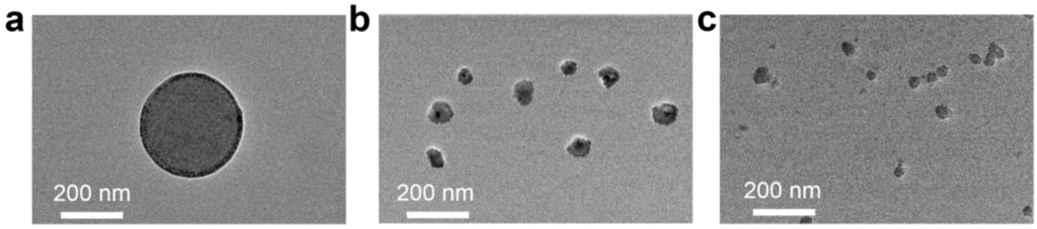

Supplementary Figure 22. TEM image of the shrinking process for ICG@Fe/FeO-PPP nanocapsules after the irradiation of laser (808 nm, 0.3 W cm^{-2}) for 5 min (pH=6.5): (a) 0 d, (b) 2 d, (c) 7d.

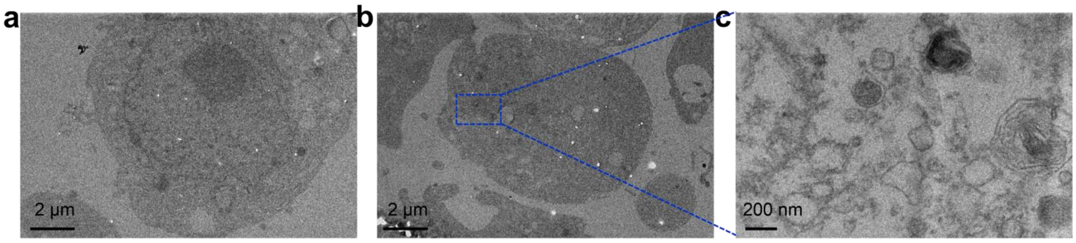

Supplementary Figure 23. Bio-TEM images of KB cells incubated with ICG@Fe/FeO-PPP nanocapsules before (a) and after 4 h (b) under the irradiation of laser (808 nm, 0.3 W cm^{-2}) for 5 min.

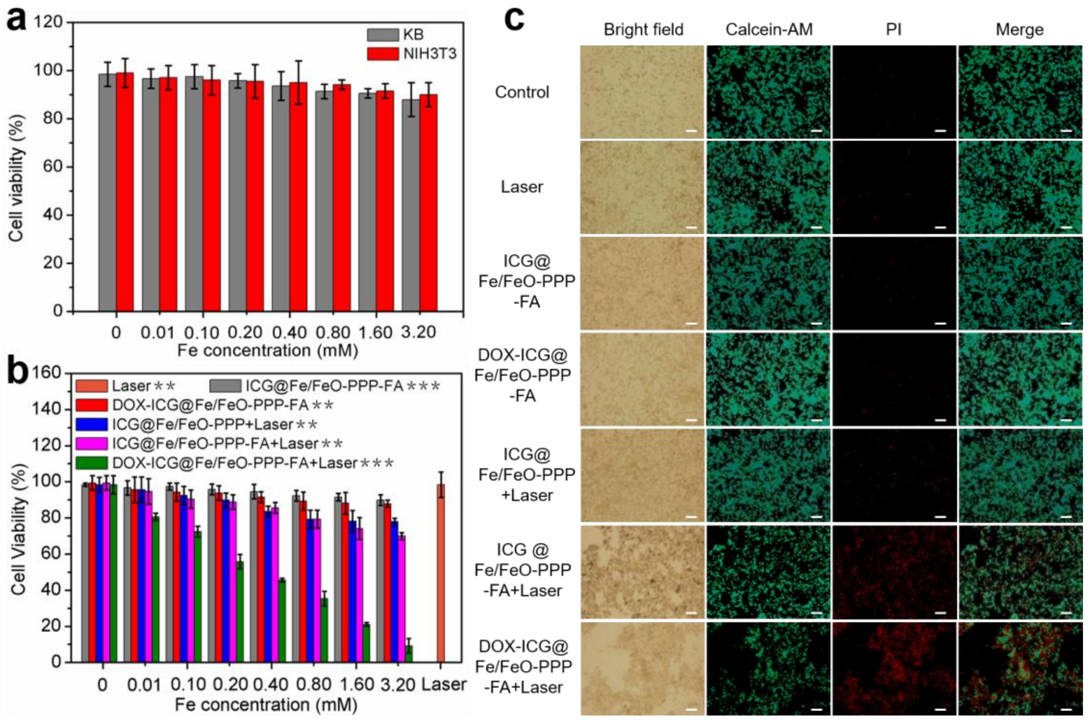

Supplementary Figure 24. *In vitro* photothermal cell ablation. (a) Viabilities of the KB (black) and NIH3T3 (red) cells determined by CCK8 assay after incubation with various concentrations of ICG@Fe/FeO-PPP for 24 h. (b) Viabilities of KB cells after incubation with various concentrations of ICG@Fe/FeO-PPP, ICG@Fe/FeO-PPP-FA and DOX-ICG@Fe/FeO-PPP-FA induced photothermal therapy under 808 nm laser irradiation (0.3 W cm^{-2}, 5 min). (c) Fluorescence microscopy images of (left) live cells, stained with Calcein-AM; (middle) necrotic or apoptotic cells, stained with PI; (right) merged, incubated with DOX-ICG@Fe/FeO-PPP-FA and irradiated with laser; KB cells incubated with ICG@Fe/FeO-PPP-FA and irradiation; KB cells incubated with ICG@Fe/FeO-PPP and irradiation; KB cells incubated with DOX-ICG@Fe/FeO-PPP-FA only; KB cells incubated with ICG@Fe/FeO-PPP-FA only; KB cells irradiated with laser only; and KB cells without any treatment, respectively (from top to bottom). Scale bars: 100 μm. In all the laser irradiation experiments, irradiation was at a power density of 0.3 W cm^{-2} for 5 min. Error bars, mean±SD. P values in (b) were calculated by Tukey's post-hoc test (**P<0.01, ***P<0.001) by comparing other groups with the last group (DOX-ICG@Fe/FeO-PPP-FA+Laser). (Error bars, mean±SD, n=3.)

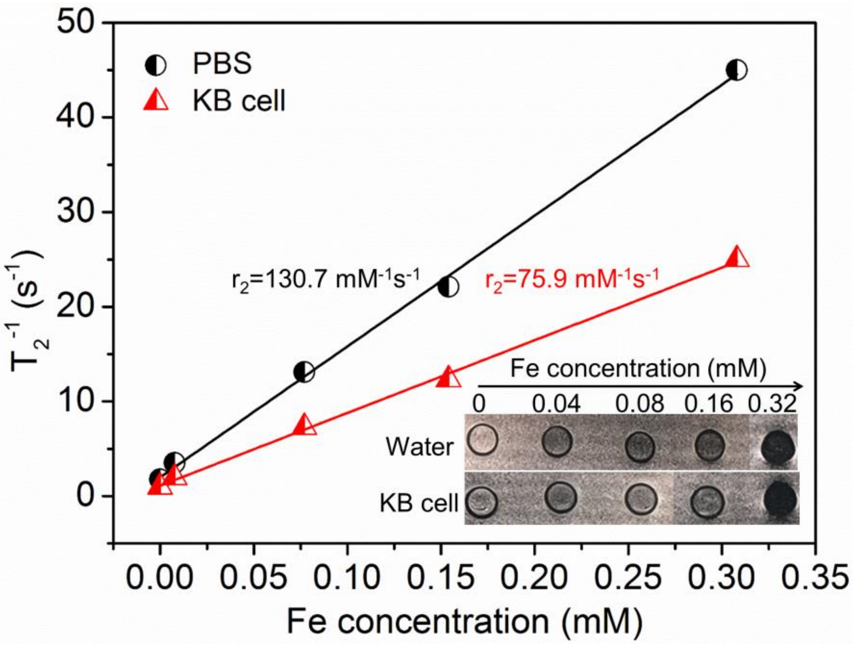

Supplementary Figure 25. Evaluation of ICG@Fe/FeO-PPP nanocapsules for *in vitro* MRI (PBS and KB cell).

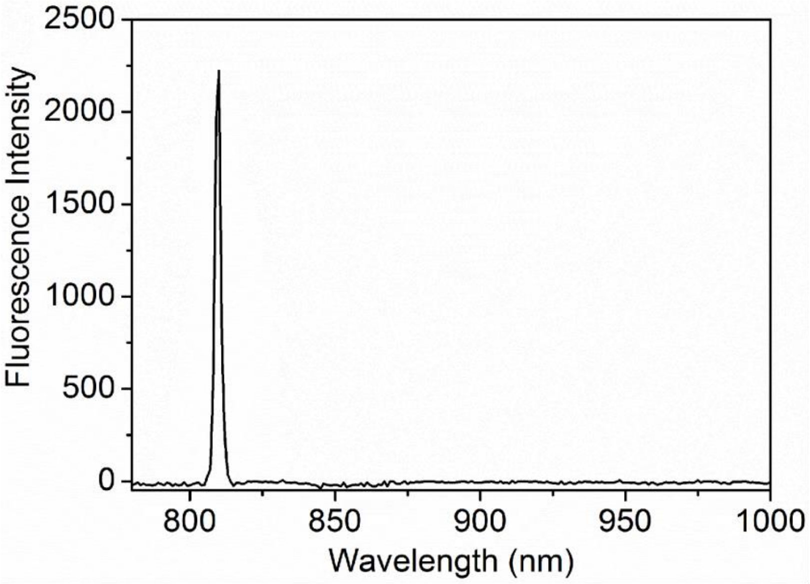

Supplementary Figure 26. Fluorescence spectrum of ICG@Fe/FeO-PPP nanocapsules ($\lambda_{ex}/\lambda_{em}$ = 780 nm/810 nm).

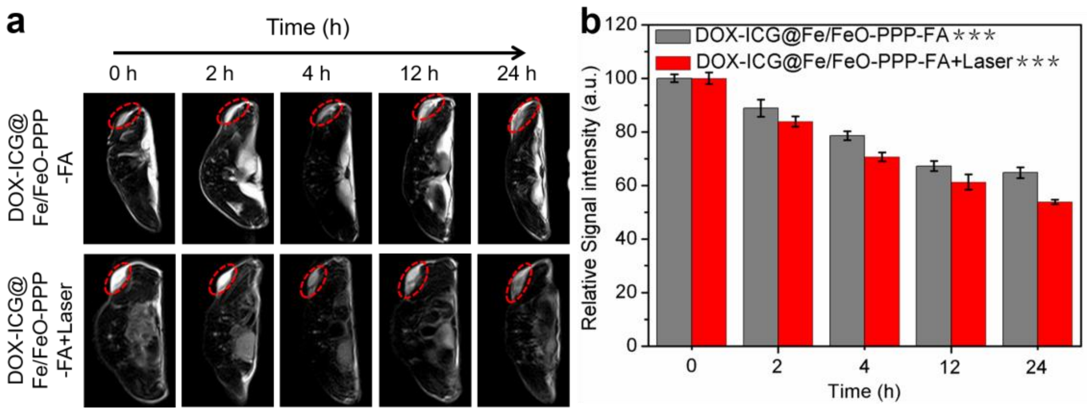

Supplementary Figure 27. (a) Real-time MRI of KB tumor-bearing mice after intravenous injection of DOX-ICG@Fe/FeO-PPP-FA nanocapsules without and with the irradiation of laser (808 nm, 0.3 W cm^{-2}, 5 min) respectively. (b) The relative MRI signal intensities changing at the tumor site respectively. P values in (b) were calculated by Tukey's post-hoc test (**P<0.01, ***P<0.001) by comparing DOX-ICG@Fe/FeO-PPP-FA group with the DOX-ICG@Fe/FeO-PPP-FA+Laser group. (Error bars, mean±SD, n=5.)

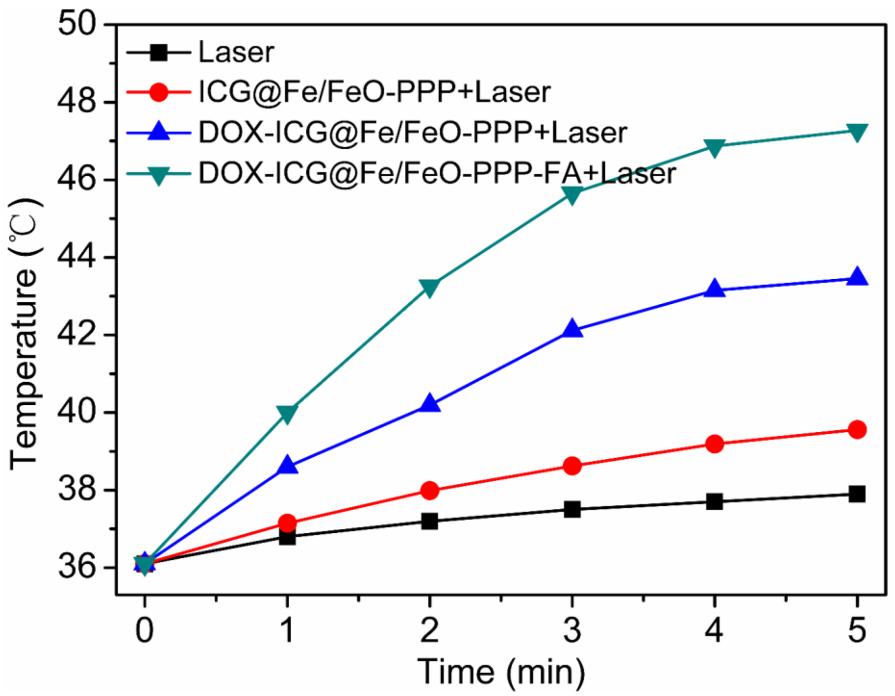

Supplementary Figure 28. Temperature change curves of tumor-bearing mice at different time points in the different treatment groups (Laser: 808 nm, 0.3 W cm^{-2}).

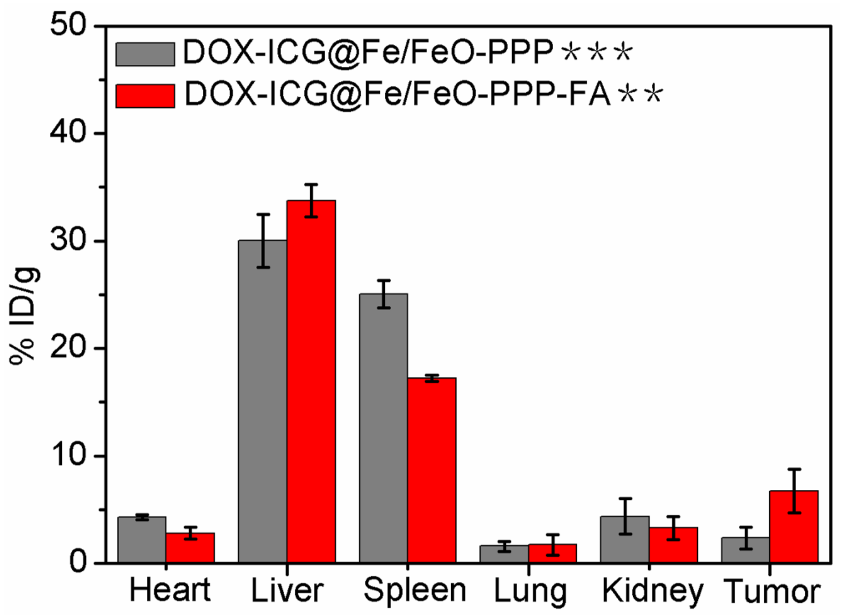

Supplementary Figure 29. The biodistribution of DOX-ICG@Fe/FeO-PPP nanocapsules and DOX-ICG@Fe/FeO-PPP-FA nanocapsules with same concentration after *i.v.* injection for 3 days by ICP-MS. P values were calculated by Tukey's post-hoc test (**P<0.01, ***P<0.001) by comparing DOX-ICG@Fe/FeO-PPP group with the DOX-ICG@Fe/FeO-PPP-FA group. (Error bars, mean±SD, n=5.)

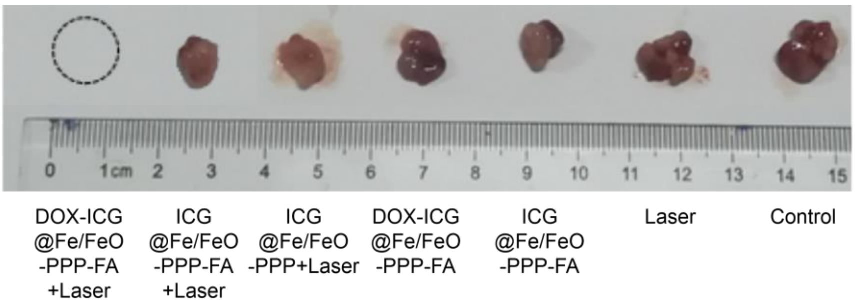

Supplementary Figure 30. Digital photos of tumor regions with different treatments after 18 days.

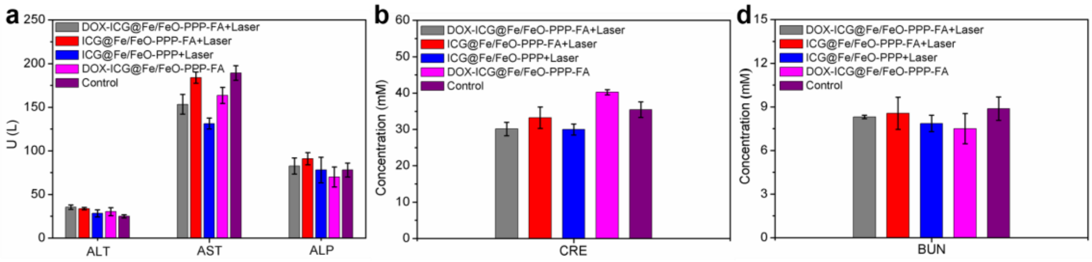

Supplementary Figure 31. *In vivo* blood biochemistry test. (a) liver function markers including ALT, ALP and AST, and kidney function markers including (b) CRE, and (c) BUN. (Error bars, mean±SD, n=5, ALT: aminotransferase; ALP: aspartate alkaline phosphatase; AST: aminotransferase; CRE: creatinine ; BUM: blood urea nitrogen.).

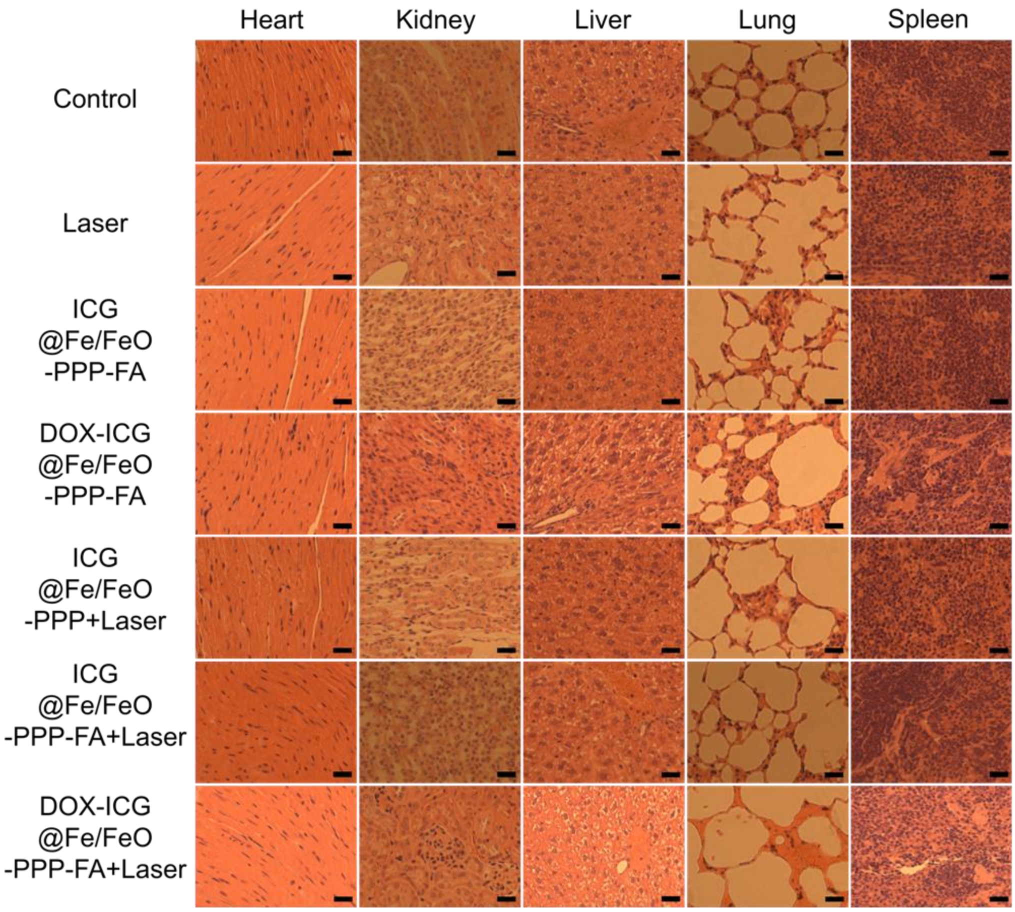

Supplementary Figure 32. H&E stained images of major organs (heart, kidney, liver, lung and spleen) collected from different groups of mice. Bars are 50 μm.
